# Dynapenic abdominal obesity and incident multimorbidity: findings from the English longitudinal study on ageing

**DOI:** 10.1007/s40520-023-02455-2

**Published:** 2023-06-05

**Authors:** Nicola Veronese, Ai Koyanagi, Pinar Soysal, Vitalba Sapienza, Francesco Saverio Ragusa, Francesco Bolzetta, Ligia J. Dominguez, Mario Barbagallo, Lee Smith

**Affiliations:** 1 Department of Health Promotion, Mother and Child Care, Internal Medicine and Medical Specialties, University of Palermo, Via del Vespro, 141, 90127 Palermo, Italy; 2 Research and Development Unit, Parc Sanitari Sant Joan de Déu, CIBERSAMISCIIIICREA, Pg. Lluis Companys 23, 08010 Barcelona, Spain; 3 Department of Geriatric Medicine, Faculty of Medicine, Bezmialem Vakif University, Istanbul, Turkey; 4 Medical Department, Geriatric Unit, Azienda ULSS (Unità Locale Socio Sanitaria) 3 “Serenissima”, Dolo-Mirano District, Dolo, Venice, Italy; 5 Faculty of Medicine and Surgery, Kore University of Enna, 94100 Enna, Italy; 6 Centre for Health, Performance, and Wellbeing, Anglia Ruskin University, Cambridge, UK

**Keywords:** Dynapenic abdominal obesity, Multimorbidity, Cohort, Risk factors

## Abstract

**Background:**

Dynapenic abdominal obesity (DAO) (i.e., impairment in muscle strength and high waist circumference) is gaining interest, as it is associated with several important adverse health outcomes. However, the association between DAO and multimorbidity is largely unclear. Thus, the aim of the present study was to investigate the association between DAO at baseline and new onset multimorbidity over ten years of follow-up.

**Methods:**

People participating in the English Longitudinal Study of Ageing were included. DAO was defined as waist circumference > 102 cm in men and > 88 cm in women, and a concomitant presence of dynapenia (handgrip strength defined as < 27 kg for men and < 16 kg for women). Multimorbidity was defined as having two or more chronic conditions. The association between DAO and incident multimorbidity was assessed using a multivariable logistic regression analysis, reporting the data as odds ratios (ORs) and their 95% confidence intervals (CIs).

**Results:**

Overall, 3302 participants (mean age: 63.4 years, males: 50.3%) without multimorbidity at baseline were followed-up for ten years. After adjusting for several variables, compared to participants without dynapenia nor abdominal obesity, the presence of abdominal obesity (OR = 1.505; 95%CI: 1.272–1.780; *p* < 0.0001) and DAO (OR = 1.671; 95%CI: 1.201–2.325; *p* = 0.002) significantly increased the risk of multimorbidity. Compared to no dynapenia nor abdominal obesity, DAO was associated with significantly higher risk for arthritis and diabetes.

**Conclusions:**

DAO was significantly associated with a higher risk of incident multimorbidity, over 10 years of follow-up. The results of our study suggest that addressing DAO can potentially decrease risk for multimorbidity.

## Introduction

Non-communicable diseases (NCDs) are now responsible for almost all global deaths [1]. It is largely known that NCDs are most common in older adults, and a substantial increase in NCDs burden is expected due to the rapid ageing population [2]. In this context, the presence of two or more chronic conditions is commonly called multimorbidity [3]. Multimorbidity is an important risk factor for several adverse health outcomes such as lower quality of life [4], increased health-care utilization [5], dementia[6], and ultimately higher risk of premature mortality [7].

In recent years, there has been increasing interest in dynapenic abdominal obesity (DAO) (i.e., impairment in muscle strength and high waist circumference)[8] as a clinical risk concept since this specific condition has been observed to be associated with several important adverse health outcomes including falls [9, 10], metabolic alterations [11], disability [12], and premature death [13], similarly to multimorbidity. Literature indicates that DAO may increase the risk for chronic diseases and consequently multimorbidity as weak muscle strength has been found to be associated with an unfavorable inflammatory profile (e.g., higher levels of C-reactive proteins and fibrinogen) [14], while the metabolic risk associated with obesity is more strongly associated with central rather than peripheral fat distribution [15]. Therefore, it is possible for people with DAO to be at particularly high risk for multimorbidity, but there are currently no longitudinal studies on this topic, that can better elucidate the potential role of DAO as a risk factor for multimorbidity.

Given this background, the aim of the present study was to investigate the association between DAO at baseline and new onset multimorbidity over 10 years of follow-up in a large representative sample of the English older adult population, while also exploring the association between DAO and single incident chronic medical conditions.

## Materials and methods

### Study population

This study is based on data from six waves (from wave 2 to wave 7) of the English Longitudinal Study of Ageing (ELSA). The ELSA study is a prospective and nationally representative cohort of men and women living in England [16]. Wave 2 (baseline survey) was conducted between 2004 and 2005, and wave 7 between 2014 and 2015. Other waves took place every two years between these dates. The ELSA was approved by the London Multicenter Research Ethics Committee (MREC/01/2/91). Informed consent was obtained from all participants.

### DAO (exposure variable)

Dynapenia was defined using the criteria proposed by the revised European consensus on the definition and diagnosis of sarcopenia [17], i.e., having weak handgrip strength defined as < 27 kg for men and < 16 kg for women (using the average value of three handgrip measurements of the dominant hand) [17]. We used mean values instead of maximum, owing to previous literature showing a good agreement between these two parameters and that in the ELSA study mean values of handgrip strength are usually employed. [18–20] Abdominal obesity was defined using the traditional cut-offs of 102 cm in men, 88 cm in women [21]. All respondents were eligible to have their waist and hip measurements taken, unless they were chairbound or had a colostomy or ileostomy. The waist circumference was evaluated twice; if the second measurement differed from the first by 3 cm or more, the nurse was given an error message and took a third measurement. The measurement of the waist was made with the participant standing. DAO was then defined as the combination of these two entities.

### Multimorbidity (outcome)

Information on the presence of medical conditions was collected by self-report on doctor diagnosed high blood pressure, diabetes, cancer, lung disease, heart conditions, stroke, psychiatric conditions, arthritis, asthma, high cholesterol levels, cataracts, Parkinson’s disease, hip fracture, Alzheimer’s disease, and other dementias. The total number of chronic conditions was then summed and multimorbidity was defined as ≥ 2 chronic conditions, in line with previously used definitions [22–24]. The presence of multimorbidity was ascertained at the baseline (wave 2) and then at wave 3, 4, 5, 6, and 7.

### Covariates

The selection of covariates was based on their previously reported associations with the exposure (DAO) and outcome (multimorbidity) [25] and included the following: age (in years, as continuous variable); sex; years of education (considered as a continuous variable); ethnicity (whites vs. non-whites); marital status (categorized as married, partnered, separated, divorced, widowed, never married); smoking status (ever vs. never); alcohol drinking (categorized as yes or no); physical activity level categorized as sedentary, low, moderate or high according to a question about the frequency with which they did moderate exercise in the previous week (e.g., gardening, cleaning the car, walking at a moderate pace, dancing, floor or stretching exercise).

### Statistical analyses

The data were weighted using the person-level longitudinal weight, core sample, wave 2 (http://www.ifs.org.uk/ELSA). Means and standard deviations (SD) were used to describe quantitative measures, while percentages and counts were used for categorical variables. Characteristics of the study participants at baseline were compared according to the presence of multimorbidity during follow-up, using Chi-squared or Fisher exact tests for categorical variables, and independent T-test for continuous variables.

The analyses were restricted to those who did not have multimorbidity at baseline to better evaluate the prospective risk of this condition among people initially free of multimorbidity. The association between the exposure variable at baseline (categorized as no dynapenia nor abdominal obesity [reference], only dynapenia, only abdominal obesity, and DAO) and incident multimorbidity was assessed using univariable and multivariable logistic regression analysis and reported as odds ratios (OR) and 95% confidence intervals (95% CI). The multivariable analysis adjusted for age, sex, alcohol drinking, educational level, ethnicity, marital status, smoking status, physical activity level, and presence of one chronic condition at baseline. The covariates used for analysis were initially chosen based on the previous literature. These variables were included in the final model if they were significantly different between people with and without multimorbidity during the follow-up in univariate analyses, using a conservative *p*-value of < 0.10. We also explored the association between DAO and incident single conditions, adjusted for all covariates, after removing the participants with that specific condition at baseline.

All statistical tests were two-tailed, and a *p*-value < 0.05 was considered statistically significant. All analyses were performed using SPSS 26.0 version software.

## Results

**Fig. 1:**
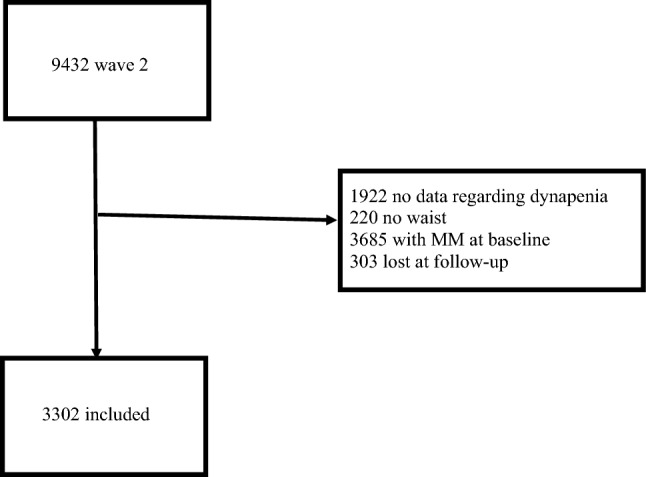
Flow chart of the study

Figure 1 shows the inclusion flow-chart for this study. At the baseline evaluation (wave 2), 9432 participants were initially considered. We subsequently removed 1922 participants since no data regarding dynapenia were available, 220 had no data regarding waist circumference, and 3685 already had two or more medical conditions at the baseline. Moreover, for 303 participants, no data regarding incident multimorbidity were available, finally leaving 3302 eligible participants for the analysis on DAO and multimorbidity.

The 3302 participants had a mean age of 63.4 ± SD 8.9 years (range: 52–90), with an approximately equal distribution of males and females (50.3%, male). During the 10-year follow-up, 1810 (55.0% of the initial population) developed multimorbidity and these people were significantly older, less educated, more frequently smokers, and less likely to consume alcohol than those without multimorbidity. Moreover, incident multimorbidity (vs. no incident multimorbidity) was associated with a lower prevalence at baseline of high physical activity level (Table 1). Among the conditions considered at the baseline, a higher prevalence of high blood pressure, diabetes, lung and heart diseases, and arthritis was observed in people with multimorbidity compared to those without. Finally, a higher prevalence of dynapenia, abdominal obesity, and DAO was observed in participants with incident multimorbidity compared to those without this condition during follow-up (Table 1).

**Table 1:** Baseline characteristics of participants who developed multimorbidity (or did not) during 10-year follow up

Characteristic	All participants (*n* = 3288)	No incident MM (*n* = 1,478)	Incident MM (*n* = 1,810)	*P*-value^a^
Female sex (%)	49.7	48.4	50.7	0.28
Age (years)	63.3 (8.9)	59.6 (6.4)	64.6 (8.7)	< 0.0001
Whites (%)	98.1	98.5	98.1	0.44
Years of education	8.2 (6.8)	9.9 (6.2)	7.8 (6.8)	< 0.0001
Ever smoking (%)	60.2	57.0	61.4	0.03
Alcohol drinking (%)	92.8	95.0	92.3	0.01
High physical activity level (%)	25.7	35.1	21.7	< 0.0001
High blood pressure (%)	16.1	7.3	21.4	< 0.0001
Diabetes (%)	1.2	0.5	1.7	0.01
Cancer (%)	2.7	2.1	3.1	0.12
Lung disease (%)	1.4	0.6	1.7	0.02
Heart conditions (%)	4.1	2.4	5.0	0.002
Stroke (%)	0.4	0.1	0.6	0.11
Psychiatric conditions (%)	2.8	2.2	3.4	0.10
Arthritis (%)	13.9	7.6	18.5	< 0.0001
Asthma (%)	4.2	2.3	5.7	< 0.0001
High cholesterol levels (%)	5.4	5.2	5.7	0.57
Cataracts (%)	3.1	2.3	3.3	0.16
Parkinson’s disease (%)	0.2	0.0	0.2	0.32
Hip fracture (%)	0.2	0.0	0.3	0.99
Dementia (%)	0.1	0.0	0.1	0.99
Only dynapenia (%)	11.1	6.9	11.4	< 0.0001
Only abdominal obesity (%)	36.3	32.3	39.4	
Dynapenic abdominal obesity (%)	8.3	3.6	10.6	

*SD* standard deviation, *MM* multimorbidity, Data are reported as percentages or as means with standard deviations. All data were obtained at the baseline evaluation. ^a^*P*-value was estimated by Chi-squared test and independent Student *T*-test for categorical and continuous variables, respectively

Table 2 shows the association between dynapenia, abdominal obesity, and DAO at baseline and multimorbidity during follow-up. Compared to participants without dynapenia and abdominal obesity, participants with only dynapenia did not report any significant risk of multimorbidity during follow-up (*p* = 0.806), whilst the presence of abdominal obesity (OR = 1.505; 95%CI: 1.272–1.780; *p* < 0.0001) and DAO (OR = 1.671; 95%CI: 1.201–2.325; *p* = 0.002) significantly increased the risk of multimorbidity. Other risk factors which were significantly associated with incident multimorbidity were older age, female sex, fewer years in education, and those separated (versus married) (Table 2).

**Table 2:** Prospective association between dynapenia, abdominal obesity, or both at baseline and multimorbidity during follow-up, estimated by multivariable logistic regression

Parameter	Sample size	Odds ratio	95% Lower CI	95% Higher CI	*p*-value
Only dynapenia	365	0.97	0.73	1.28	0.81
Only abdominal obesity	1193	1.51	1.27	1.78	< 0.0001
Dynapenic abdominal obesity	273	1.67	1.20	2.33	0.002
Age (years)	3288	1.07	1.06	1.09	< 0.0001
Male sex	1653	0.78	0.64	0.96	0.02
Alcohol drinking	2791	0.86	0.56	1.33	0.494
Years of education	3288	0.98	0.97	1.00	0.02
Marital status (married as reference)					
Partnered	94	0.61	0.34	1.07	0.08
Separated	46	0.39	0.16	0.94	0.04
Divorced	235	1.13	0.76	1.68	0.54
Widowed	382	1.02	0.67	1.54	0.93
Never married	149	0.70	0.42	1.18	0.18
Ever smoking	1978	1.23	0.93	1.62	0.15
Physical activity level (sedentary as reference)					
Low	549	1.93	0.70	5.28	0.20
Moderate	1835	1.70	0.64	4.54	0.29
High	844	1.11	0.41	2.99	0.84
Whites	3244	1.66	0.66	4.15	0.28

*OR* Odds ratio, *CI* Confidence interval. Model is mutually adjusted for all variables in the Table Incident multimorbidity (outcome) was assessed during the follow-up period and referred to those that occurred since the baseline evaluation among people without multimorbidity at baseline

Table 3 shows the association between dynapenia, abdominal obesity, or DAO, at baseline and single medical conditions during follow-up. Compared to people without dynapenia or abdominal obesity, the presence of only dynapenia significantly increased the risk of diabetes (OR = 2.44; 95%CI: 1.21–4.91; *P* = 0.001), whilst the presence of only abdominal obesity increased the risk of high blood pressure (OR = 1.43; 95%CI: 1.15–1.78; *P* = 0.001), diabetes (OR = 4.56; 95%CI: 2.99–6.95; *P* < 0.0001), arthritis (OR = 1.48; 95%CI: 1.18–1.86; *P* = 0.001), and high cholesterol levels (OR = 1.24; 95%CI: 1.03–1-49; *P* = 0.02). DAO increased the risk of diabetes (OR = 5.87; 95%CI: 3.13–11.03; *P* < 0.0001) and arthritis (OR = 1.92; 95%CI: 1.27–2.92; *P* = 0.002) (Table 3).

**Table 3:** Prospective association between dynapenia, abdominal obesity, or both at baseline and single medical condition during follow-up, estimated by multivariable logistic regression

Condition	Sample size included at the baseline	Incident cases	Percentage	Only dynapenia	Only abdominal obesity	Dynapenic abdominal obesity
High blood pressure	2761	1133	41.0	0.85 (0.59–1.24) *P* = 0.41	1.43 (1.15–1.78) *P* = 0.001	1.44 (0.97–2.13) *P* = 0.07
Diabetes	3249	225	6.9	2.44 (1.21–4.91) *P* = 0.001	4.56 (2.99–6.95) *P* < 0.0001	5.87 (3.13–11.03) *P* < 0.0001
Cancer	3200	356	11.1	0.52 (0.30–1.09) *P* = 0.23	0.91 (0.67–1.23) *P* = 0.53	0.92 (0.54–1.57) *P* = 0.77
Lung diseases	3243	141	4.3	1.01 (0.48–2.13) *P* = 0.98	0.68 (0.40–1.15) *P* = 0.15	1.63 (0.74–3.61) *P* = 0.23
Heart conditions	3152	505	16.0	1.28 (0.84–1.95) *P* = 0.24	1.30 (0.99–1.72) *P* = 0.06	1.15 (0.71–1.86) *P* = 0.58
Stroke	3276	119	3.6	0.96 (0.50–1.87) *P* = 0.91	0.67 (0.39–1.15) *P* = 0.15	0.56 (0.24–1.30) *P* = 0.18
Psychiatric conditions	3196	184	5.7	1.20 (0.54–2.67) *P* = 0.66	0.79 (0.46–1.36) *P* = 0.40	1.11 (0.43–2.92) *P* = 0.83
Arthritis	2831	997	35.2	1.34 (0.91–1.99) *P* = 0.13	1.48 (1.18–1.86) *P* = 0.001	1.92 (1.27–2.92) *P* = 0.002
Asthma	3149	220	7.0	0.91 (0.35–2.35) *P* = 0.84	1.07 (0.63–1.85) *P* = 0.80	2.22 (0.98–5.03) *P* = 0.06
High cholesterol levels	3109	1117	35.9	0.88 (0.65–1.21) *P* = 0.44	1.24 (1.03–1-49) *P* = 0.02	0.79 (0.55–1.14) *P* = 0.21
Cataracts	3182	864	27.2	0.84 (0.61–1.17) *P* = 0.31	1.12 (0.91–1.38) *P* = 0.28	1.20 (0.85–1.71) *P* = 0.30
Parkinson’s disease	3280	41	1.3	0.21 (0.03–1.58) P = 0.13	0.84 (0.39–1.81) P = 0.66	0.79 (0.20–3.06) P = 0.73
Hip fracture	1862	61	3.3	0.87 (0.32–2.39) *P* = 0.78	1.05 (0.46–2.41) *P* = 0.91	0.29 (0.07–1.24) *P* = 0.09
Alzheimer’s disease	3286	31	1.0	0.90 (0.25–3.31) *P* = 0.88	0.23 (0.05–1,18) *P* = 0.08	1.10 (0.29–4.20) *P* = 0.89
Dementia	3284	79	2.4	0.37 (0.14–1.03) *P* = 0.08	0.64 (0.31–1.33) *P* = 0.23	0.76 (0.31–1.33) *P* = 0.54

Incident chronic conditions (outcome) were assessed during the follow-up period and referred to those that occurred since the baseline evaluation without having that specific condition at the baseline. Results are reported as odds ratios with 95% confidence intervals and their *p*-values, fully-adjusted for age, sex, alcohol drinking, educational level, ethnicity, marital status, smoking status, physical activity level. In all the analyses, participants with no dynapenia nor abdominal obesity at the baseline were taken as reference

## Discussion

In this large nationally representative sample of UK adults, we found that the presence of abdominal obesity and DAO significantly increased the risk of multimorbidity over ten years of follow-up. DAO was significantly associated with incident multimorbidity, whilst dynapenia was not. Moreover, DAO further increases the risk of multimorbidity during the follow-up when compared to the presence of only abdominal obesity. In terms of the individual chronic conditions, compared to no dynapenia nor abdominal obesity, DAO was associated with significantly higher risk for arthritis and diabetes. To the best of the authors’ knowledge, this is the first study, with a prospective design, on the association between DAO and multimorbidity.

A first relevant epidemiological finding of our study is that approximately half of the population included at baseline had two or more medical conditions and were therefore excluded from our analyses. Considering that the mean age of the participants included in our study was approximately 63 years, we can consider that a consistent number of young-old people are already comorbid indicating a population that is in poorer health than expected [26]. Moreover, our study showed that during the follow-up period of 10 years, a large proportion of the population became multimorbid, reinforcing the importance of studies that can identify risk factors for this condition, such as DAO, which is potentially reversible. Several mechanisms can explain the association between DAO and incident multimorbidity. First, DAO is characterized by dynapenia and central adiposity, and both these conditions are associated with higher serum inflammatory markers [14, 27]. In this regard, cross-sectional studies have found a significant association between inflammatory markers and sarcopenia or dynapenia [28]. Some inflammatory cytokines, such as interleukin (IL)-6 or tumor necrosis factor (TNF)-α, seem to increase skeletal muscle breakdown, slow protein synthesis, and inhibit plasma concentrations of insulin-like growth factors that could impair muscle anabolic processes [29, 30]. Moreover, the presence of central adiposity could further impair the immune response [31], and adipose tissue is now considered an endocrine organ that can produce and secrete several inflammatory molecules [32]. Dysregulation of inflammatory responses can lead to the development of several physical medical conditions by affecting immunity, for example. [33]

Regarding DAO and individual chronic conditions, we found a positive and significant association between DAO with arthritis and diabetes, during ten years of follow-up. The association between DAO and higher risk of arthritis may be mainly explained by central adiposity. Indeed, in the present study central obesity alone was also associated with a higher risk of arthritis and obesity per se is a well-known risk factor for arthritis [34]. Moreover, the link between DAO and diabetes is also likely mainly driven by central obesity. It is known that abdominal obesity increases the risk of diabetes via changes in function of adipose tissue, specifically, increased release of free fatty acids and abnormalities in adipokine secretion resulting in insulin resistance [35]. Furthermore, abdominal obesity is associated with a higher insulin resistance level, a key marker of diabetes [36]. Our study showed that dynapenia at baseline was also associated with a higher risk of diabetes, confirming other findings indicating a significant association between sarcopenia and diabetes, independently from obesity, which could be explained by increases in insulin resistance level [37].

We believe that our epidemiological findings could have relevant clinical implications since they suggest that interventions to manage DAO could potentially prevent multimorbidity. In this regard, while pharmacological interventions are still not available, physical activity and nutrition interventions could be most appropriate [38]. For example, resistance training could be particularly important since this kind of intervention could decrease body fat mass and at the same time increase muscle strength, and muscle performance [39]. Of importance, our study indicated that low physical activity level is another independent risk factor for incident multimorbidity during the follow-up period. Similarly, nutritional interventions are of importance to reach an adequate weight loss and therefore improve inflammation and insulin resistance [40]. However, since hypocaloric diets may lead to a loss in muscle mass, the combination of physical activity in DAO is of importance.

The findings of our study must be interpreted in light of the study’s limitations. First, most of the study variables, including those regarding multimorbidity were self-reported and this could introduce recall bias, however, for several medical conditions in the ELSA study, a good agreement between self-reported and medical records have been observed [41]. However, other studies have reported that a large variability may exist with a low agreement between medical records and self-reported information. However, self-reported medical data may be sufficient for ruling out history of a particular condition [42]. Second, while our list of chronic conditions probably included most important conditions that occur in late life, it is possible for the results to have differed with a list of different chronic conditions. Third, the population was largely Caucasian, since the waist circumference cut-off is ethnic-specific, incident MM may differ in other populations. Therefore, studies from other ethnic groups are needed which may or may not reinforce the present findings. Finally, some relevant risk factors, such as the use of medications, owing to data not being available, were not included in the present analyses and could modify the association that we found in our study.

In conclusion, in the present study including a large representative sample of middle-aged and older UK adults, DAO was significantly associated with a higher risk of incident multimorbidity, compared to no dynapenia nor abdominal obesity, over ten years of follow-up. Interventions to prevent or manage DAO may also aid in the prevention of (multiple) chronic conditions that are typical of DAO such as arthritis and diabetes, usually associated with a poor quality of life. Future intervention studies are, however, needed before concrete recommendations can be made.

## Data Availability

The original contributions presented in the study are included in the article; further inquiries can be directed to the corresponding author.
